# The feasibility and safety of cryoballoon ablation in an elderly patient with cor triatriatum sinister and persistent atrial fibrillation: A case report and a review

**DOI:** 10.1016/j.hrcr.2025.06.025

**Published:** 2025-06-26

**Authors:** Abay Bakytzhanuly, Yerkin Azatov, Zhandos Esilbaev, Yerlan Turubayev, Omirbek Nuralinov

**Affiliations:** 1 Interventional Arrhythmology Department, “University Medical Center” Corporate Fund, Astana, Kazakhstan; 2 Cardiology Department Number 2, “University Medical Center” Corporate Fund, Astana, Kazakhstan

**Keywords:** Cor triatriatum sinister, Cryoballoon ablation, Atrial fibrillation, Congenital heart disease, Electrophysiology

**Key Teaching Points**

• Cryoballoon ablation can be successfully performed in patients with cor triatriatum sinister, despite the unique anatomic challenges posed by the left atrial membrane. This case highlights the importance of preprocedural imaging to guide transseptal access and pulmonary vein isolation.
• Intracardiac echocardiography and 3-dimensional mapping are crucial in navigating complex atrial anatomy during catheter ablation in patients with congenital heart disease, ensuring safe and effective lesion delivery.
• Electrophysiologists should be aware of the anatomic variations in cor triatriatum sinister that may affect catheter maneuverability. Individualized procedural planning is essential to optimize outcomes and minimize complications.

## Introduction

Cor triatriatum is a rare congenital heart malformation in which the atrium is subdivided by a septum. The latter is usually a thin fibromuscular membrane stretching in the left or right atrium, resulting in a triatrial heart. Communication between 2 atrial compartments occurs through the fenestration in the fibromuscular septum.^1^ This heart defect is classified into 3 groups: cor triatriatum dextrum, cor triatriatum sinister (CTS), and cor triatriatum intermedium. In cor triatriatum dexter, the sinus venosus valve persists, separating the right atrium into 2 compartments. The left cor triatriatum is observed more frequently than the right cor triatriatum, with 0.1% to 0.4% and 0.025% of all congenital heart diseases, respectively. Cor triatriatum intermedium is only a theoretical type that may persist according to a persistent interseptovalvular space, and no real case reports have been observed.^2^ In CTS, the fibromuscular septum divides the left atrium (LA) into posterior-superior and anterior-inferior compartments. The superior compartment contains only pulmonary veins (PVs), but the LA appendage (LAA) and mitral annulus are obligatory parts of the inferior compartment. This anatomic allocation is key in differential diagnosis with other heart malformations.^3^ The fenestration location depends on the patient, symptoms similar to mitral stenosis, and depends on the degree of the obstruction: type 1 with no communication between the LA chambers, type 2 with small communication, and type 3 with large communication (>1 cm). Type 3 patients without any congenital heart diseases and comorbidities are generally asymptomatic.^4^ However, in life, they may experience hypertension, atrial fibrillation (AF), and other diseases, which will lead to checkups.

## Case report

**Figure 1:**
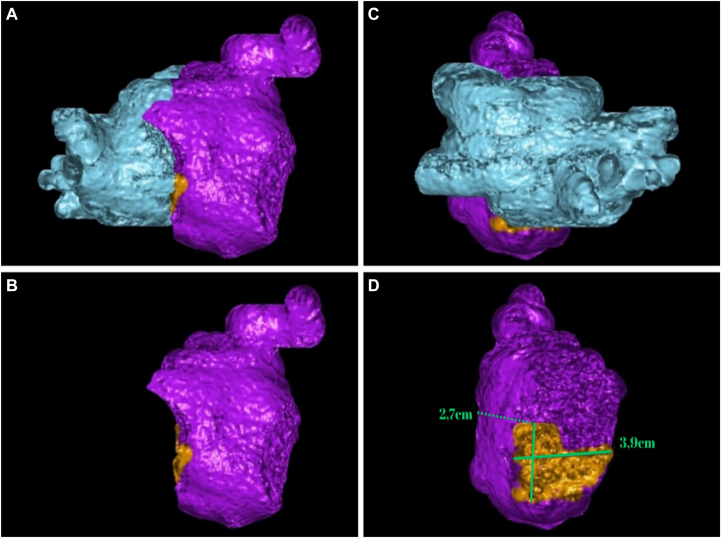
Computed tomography visualization of the LA. **A–B:** RAO projection of the LA with its compartments. The mitral annulus and the LAA are in the inferior-anterior compartment (*purple*) of CTS. **C:** PA projection of LA with 4 pulmonary veins in the posterior-superior compartment (*blue*) of CTS. **D:** PA projection of LA with removed posterior-superior compartment and demonstration of fibromuscular membrane (*orange*), diameter 2.7 × 3.9 cm. CTS = cor triatriatum sinister; LA = left atrium; LAA = left atrial appendage; PA = posterior-anterior; RAO = right anterior oblique.

A 70-year-old woman with a body mass index of 34.05 kg/m^2^ presented to our clinic with a history of hypertension and experienced dyspnea and palpitations for 5 months. The electrocardiogram (ECG) and Holter ECG registered AF. The therapy at the moment of presentation to our clinic was amiodarone, rivaroxaban, and valsartan. According to AF progression, the patient was hospitalized for a checkup. Transthoracic echocardiography revealed CTS for the first time, and additional computed tomography (CT) was performed to visualize the anatomy of the congenital heart disease and exclude LAA thrombosis. All PVs were typically connected to the LA and were in the posterior-superior LA compartment. The LAA and mitral valve annulus were in the anterior-inferior compartment (Figure 1), and LAA thrombosis was excluded. According to transthoracic echocardiography, the patient had no anomalies except for CTS. The LA dimensions were volume of 46.5 mL, indexed volume of 25.41 mL/m^2^, diameter of 3.8 cm, length of 6.3 cm, and width of 2.8 cm, and the diameter of the fibromuscular membrane was 3.5 cm (2.7 × 3.9 cm by CT) with a 1.8 cm fenestration orifice (type 3). The volume of the superior-posterior compartment of the LA by CT was 45 mL, whereas the volume of the anterior-inferior compartment was 83 mL.

The large fenestration in CTS did not influence the patient’s quality of life and hemodynamics; therefore, surgical correction was not recommended. The heart team decided to perform catheter ablation for AF. In our clinical protocol, we perform for all first AF cases cryoballoon PV isolation (PVI) and radiofrequency catheter ablations in redo procedures. The preoperative assessment revealed that all blood analyses, including electrolytes, hepatic and renal functions, thyroid function, coagulation status, lipid profile, and blood counts, were within normal range.

**Figure 2:**
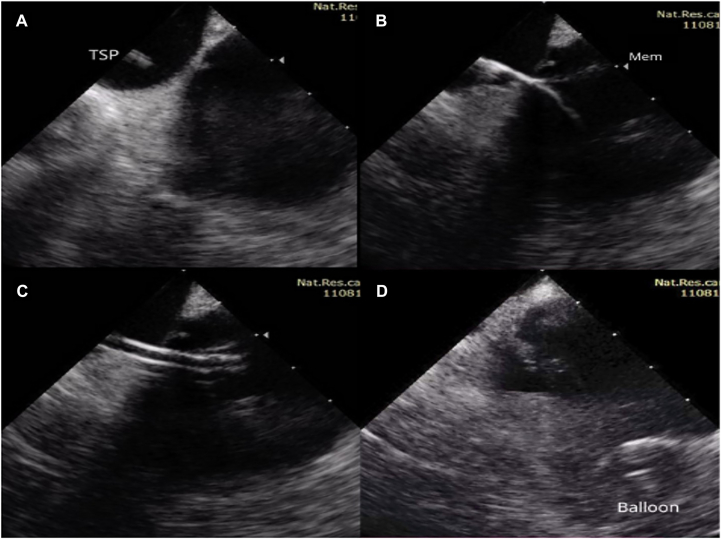
Two-dimensional intracardiac ultrasound modeling images. **A:** The transeptal puncture needle is positioned at the posterior interatrial septum. **B:** Transeptal puncture and guidewire introduction into LA. **C:** The FlexCath Steerable Sheath was introduced to the LA. **D:** Cryoballoon ablation of the left superior pulmonary vein. Balloon = inflated cryoballoon; LA = left atrium; Mem = fibromuscular membrane; TSP = transeptal puncture needle.

After obtaining an informed consent, the patient was referred for coronary angiography and cryoballoon PVI. The coronary angiography excluded coronary artery pathology. The left femoral vein approach was used for intracardiac echocardiography (ICE) (AcuNav). The right femoral vein was punctured twice for placement of a decapolar diagnostic electrode in the coronary sinus position and a sheath Swartz SL0 (Abbott) with a Brockenbrough needle for transseptal puncture (TSP). Under fluoroscopy and ICE guidance, TSP was performed more posteriorly (Figure 2). According to the ICE, the interatrial septum was thick and rigid; therefore, a needle stylet was used to perform TSP (the “mosquito” technique). The total amount of heparin was 10,000 IU. During the procedure, the activated clotting time was >300 seconds.

Afterward, LA contrast angiography was performed for a clear visualization of PVs and correct TSP. All PVs underwent single cryoballoon application for 240 seconds, reaching the temperature from −45 °C to −56 °C with isolation in 45, 55, 48, and 57 seconds (left superior, left inferior, right inferior, and right superior, respectively). To prevent phrenic nerve palsy, the decapolar catheter was placed on the lateral wall of the superior vena cava during right PV cryoablation. After the accomplishment of the PVI, the Z-type suture was used for hemostasis. On the second day after PVI, the patient was discharged. A 6-month follow-up showed a stable sinus rhythm on Holter ECG and no patient complaints.

## Discussion

The presence of a fibromuscular membrane complicates the mapping or catheterization maneuvers of CTS.^5^^,^^6^ Given that cor triatriatum was described for the first time, only 8 cases of catheter ablation of AF were reported, including 1 reisolation owing to AF recurrence (Table 1).^5^, ^6^, ^7^, ^8^, ^9^, ^10^, ^11^, ^12^ All cases with AF were in patients with CTS, and all patients were men with a mean age of 62.2 ± 7.05 years. Five patients had paroxysmal AF, 1 long-standing persistent AF, and 2 persistent AF.

**Table 1:** Catheter ablation of AF in patients with cor triatriatum sinister

Case #	First author	Published year	Patients’ age	Patients’ gender	Type	Associated congenital diseases	Type of AF	TSP	Procedure	Index procedure	Outcome
**1**	Yamada^5^	2008	66	M	3	None	Paroxysmal	Fluoroscopy guided	RFA	PVI	Recurrence after 3 mo
**2**	Yamada^6^	2009	66	M	3	None	Paroxysmal	Fluoroscopy guided	RFA	Redo PVI	AF-free, off AAD at 6 mo
**3**	Bhatia^7^	2010	72	M	3	None	Paroxysmal	ICE guided	RFA	PVI + CTI	AF-free, off AAD at 6 mo
**4**	Gavin^8^	2011	64	M	3	None	Long-standing, persistent	TEE guided	RFA	PVI + LA roof + MI + CFAE	AF-free, off AAD at 7 mo
**5**	Fukumoto^9^	2012	58	M	3	None	Paroxysmal	ICE guided	RFA	PVI	AF-free, off AAD at 24 mo
**6**	Tokuda^10^	2016	57	M	3	None	Persistent	ICE guided	RFA	PVI + LAPWI	AF-free, off AAD, at 12 mo
**7**	Borne^11^	2016	51	M	3	None	Paroxysmal	ICE guided	RFA	PVI + LA roof + MI + CT	AF-free, off AAD, at 12 mo
**8**	Morishima^12^	2020	56	M	3	None	Persistent	ICE guided	RFA	PVI + SVCI	AF-free, off AAD, at 36 mo
**9**	Present case	2024	70	F	3	None	Persistent	ICE guided	CRYO	PVI	AF-free for 6 mo

AAD = antiarrhythmic drug; AF = atrial fibrillation; CFAE = complex fractionated atrial electrograms; CRYO = cryoablation; CT = computed tomography; CTI = cavotricuspid isthmus; F = female; ICE = intracardiac echocardiography; LA = left atrium; LAPWI = left atrial posterior wall isolation; M = male; MI = mitral isthmus; PVI = pulmonary vein isolation; RFA = radiofrequency ablation; SVCI = superior vena cava isolation; TEE = transesophageal echocardiogram; TSP = transseptal puncture.

**Figure 3:**
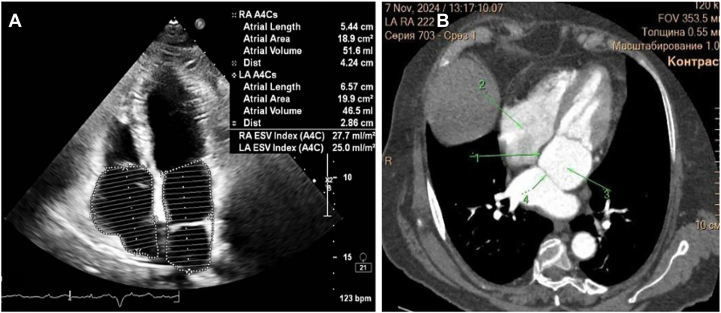
**A:** Transthoracic echocardiography 4-chamber position. **B:** Computer tomography of the heart with contrast. 1 = interatrial septum; 2 = right atrium; 3 = anterior-inferior compartment; 4 = fibromuscular membrane.

**Figure 4:**
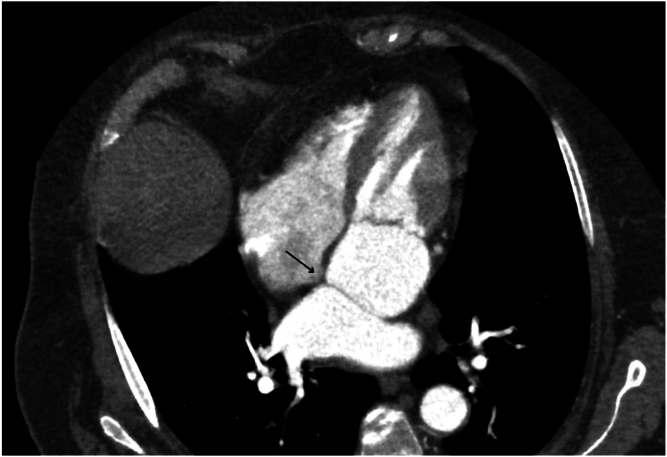
Fossa ovalis anatomy. The fibromuscular membrane attaches to the middle of the fossa ovalis, which is indicated by a *little black arrow*.

Catheter ablation of AF in patients with CTS presents us with unique anatomic and technical challenges. The existence of a fibromuscular membrane dividing the LA into 2 compartments alters the normal anatomic geometry of the LA, potentially complicating the main aspects of catheter ablation, such as TSP and catheter maneuvering (Figure 3). Despite these challenges, we successfully performed cryoballoon ablation in a 70-year-old woman with persistent AF and CTS, illustrating the safety and feasibility of this approach. A review of the existing literature demonstrates that previous reports of AF ablation in patients with CTS exclusively involved radiofrequency ablation (RFA) for point-by-point PVI (Table 1).^5^, ^6^, ^7^, ^8^, ^9^, ^10^, ^11^, ^12^ Our case represents the first documented use of cryoballoon PVI in an adult patient with CTS. The choice of cryoballoon ablation for this patient over RFA was because of its efficiency in achieving stable PVI, shorter procedural time, and lower perioperative risks (as recommended by American and European guidelines). The critical step in this case is TSP. Because the fibromuscular membrane attaches the fossa ovalis in the middle (Figure 4) and all 4 PVs are located in the superior-posterior compartment of the LA, the TSP is performed more posteriorly. Owing to the thickness and the rigidity of the interatrial septum, the “mosquito” technique was used during TSP, ensuring safe and effective access to PVs. The “mosquito” technique is commonly used in cases when TSP is challenging owing to the rigidity of the interatrial septum, and it is performed by putting a stylet inside the needle after unsuccessful TSP attempts with the needle alone. Because the stylet is thinner than the needle, it easily perforates the interatrial membrane, after which the needle can also perforate the interatrial septum. However, this technique requires the surgeon’s confidence that the needle is standing in the fossa ovalis; otherwise, serious complications can occur. In previous cases of AF ablation in CTS, the operators reported the need for additional imaging guidance, including ICE and CT, to achieve procedural success. Our case further underlines the importance of multimodal imaging in patients with CTS undergoing catheter ablation. Another interesting moment to mention is moving the FlexCath inside the posterior compartment of the LA during cryoballoon ablation. We moved the FlexCath smoothly under ICE and fluoroscopy control while standing in each PV. A slight pulling back maneuver was used while moving the FlexCath from the left inferior PV to the right inferior PV with regard to the volume of the posterior-superior compartment and more posterior TSP. A key moment to discuss is whether the fibromuscular membrane itself contributes to arrhythmogenesis in patients with AF in CTS. Previous studies have not demonstrated direct arrhythmic foci from the membrane. However, altered atrial substrate and hemodynamic changes may increase the risk of AF in patients with CTS. In our case, the fenestration between 2 compartments was more than 1 cm, suggesting minimal obstruction; however, the patient still developed AF at the age of 70 years, likely owing to progressive atrial remodeling and hypertension. Further studies are needed to identify the role of atrial anatomic variations in arrhythmogenesis.

Our case expands the existing literature by providing evidence that cryoballoon ablation is a feasible and effective approach for patients with AF in CTS. The patient’s stable sinus rhythm during the 6-month follow-up suggests the efficiency of cryoballoon ablation in achieving stable PVI comparable with RFA PVI, as presented in previously reported cases. However, long-term follow-up and larger case series would be valuable to confirm procedural safety and efficacy.

### Limitations

• The follow-up period of 6 months is relatively short, and longer-term results would be valuable.
• Our patient had persistent AF, whereas most previous cases involved paroxysmal AF. This may affect the comparisons with previous reports.
• We did not assess the electrophysiological properties of the fibromuscular membrane, which could provide insights into its role in AF pathophysiology.

## Conclusion

This case is the first documented instance of cryoballoon ablation for AF in an adult with CTS, demonstrating that PVI can be performed safely and effectively despite anatomic challenges. Our findings highlight the significance of individualized procedural planning, imaging guidance, and careful transseptal access. Further research is necessary to identify the long-term success of cryoballoon PVI in this group of patients with rare congenital anomalies.
